# Application and Research Prospect of Functional Polymer Gels in Oil and Gas Drilling and Development Engineering

**DOI:** 10.3390/gels9050413

**Published:** 2023-05-16

**Authors:** Yingrui Bai, Yuan Liu, Keqing Yang, Youming Lang

**Affiliations:** School of Petroleum Engineering, China University of Petroleum (East China), Qingdao 266580, China

**Keywords:** polymer gel, formation mechanism, drilling fluid, fracturing fluid, enhanced oil recovery

## Abstract

Polymer gel materials are formed by physically crosslinking and chemically crosslinking to form a gel network system with high mechanical properties and reversible performance. Due to their excellent mechanical properties and intelligence, polymer gel materials are widely used in biomedical, tissue engineering, artificial intelligence, firefighting and other fields. Given the current research status of polymer gels at home and abroad and the current application status of oilfield drilling, this paper reviews the mechanism of polymer gels formed by physically crosslinking and chemically crosslinking, summarizes the performance characteristics and the mechanism of action of polymer gels formed by non-covalent bonding, such as hydrophobic bonding, hydrogen bonding, electrostatic and Van der Waals interactions interactions, and covalent bonding such as imine bonding, acylhydrazone bonding and Diels-Alder reaction. The current status and outlook of the application of polymer gels in drilling fluids, fracturing fluids and enhanced oil recovery are also introduced. We expand the application fields of polymer gel materials and promote the development of polymer gel materials in a more intelligent direction.

## 1. Introduction

Polymer gel is a three-dimensional crosslinked network composed of molecules with hydrophilic (polar) and hydrophobic groups on the main chain and side chains of macromolecules, or with dissociated groups, and the solvent is fixed in the molecular network, which is a material form between liquid and solid. The research on polymer gels at home and abroad is relatively well-established; as early as the 1940s and 1950s, Flory and Stockmayer studied the phenomenon of dissolution of electrolyte gels, and in 1960, Otto Wichterle et al. discovered the first synthetic hydrogel [1]. In recent years, the research on polymer gel materials has received a lot of attention from domestic and foreign experts and scholars. Due to the previously developed polymer gels with inhomogeneous network crosslinked structures and the lack of sufficient energy dissipation mechanisms to prevent their fracture, conventional polymer gel gels usually exhibit poor mechanical strength or toughness, and therefore, the practical applications of polymer gels are severely limited. However, with the continuous development of technology, polymer gels exhibit excellent intelligence and high strength and are widely used in biomedicine, tissue engineering, artificial intelligence, etc. [2,3,4]. Polymer gels have excellent loading capacity and extended-release and adjustable physicochemical properties [5]; novel polymer gels have antimicrobial effects during wound healing and promote tissue regeneration [6]; foreign scholars have studied UHMWPE, which as a lightweight, low-friction and high-impact material for robot joints to increase energy efficiency and reduce the power consumption of robots [7].

Polymer gels are classified into physical crosslinking gels and chemical crosslinking gels. Physically crosslinking gels are formed by non-covalent bonding with each other rather than by crosslinking agents, mainly hydrophobic bonding, hydrogen bonding, electrostatic, van der Waals interactions forces and other interactions. Self-assembled, non-covalent interactions of polymer gels are weaker in strength, but they have dynamic reversibility and can achieve sol–gel mutual transformation. Chemically crosslinking gels are not easily degradable enough for environmental protection, although they have better structural stability and mechanical properties [8]. Compared with conventional gels, polymer gels have discontinuity and are better able to produce deformation and respond quickly to environmental stimuli. Therefore, with the continuous development in the field of polymer chemistry, there are new opportunities for stimuli-responsive self-healing gels [9].

Along with the development of oil exploration and development and the development of deep, ultra-deep, unconventional and low-grade hydrocarbon resources, the environment of development is becoming more and more severe. Polymer gel, due to its remarkable mechanical properties, has strong self-adaptability for sealing pores and fractures of different sizes and is widely applied in oil and gas drilling for leakage prevention, plugging, fracturing, cementing, dissection and water plugging of the formation and improving the enhanced oil recovery, etc. As such, polymeric gels are one of the most widely used chemicals in oil and gas wells [10]. According to the crosslinking form of polymer gels, they can be divided into surface pre-crosslinking gels, subsurface crosslinked gels and non-chemical crosslinking gels. Underground crosslinked gel, also known as in situ crosslinking gel, is used to solve the problem of severe leakage or large total leakage and to meet the plugging and pumping requirements of different good depths, which require stopping drilling and plugging, and high-strength block gel can be produced in the hole and crack to reach the high-intensity sealing area. Ground pre-crosslinked gel, which is formed by mixing the polymer with the crosslinking agent in the ground equipment, forms a blocking zone by an expanding, aggregation and filling effect of gel particles after the completion of crosslinking. Ground pre-crosslinked gels are not sensitive to PH, temperature, shear, salinity, etc. They can, therefore, be used in a wider range of applications. The most commonly used pre-crosslinked gels are PPGs, polymer microspheres and colloidal dispersion gels. Non-chemical crosslinked gels are polymeric aqueous solutions that undergo phase changes stimulated by changes in environmental conditions such as temperature, pH and mineralization, and the process of gel formation is reversible and can undergo a gel-solution transition, such as hydrogels. The ability of hydrogels to self-heal depends largely on dynamic crosslinking mechanisms, including reversible covalent bonds (such as imine bonds, acylhydrazone bonds and Diels–Alder reaction) and non-covalent interactions (such as hydrophobic bonding, hydrogen bonding, electrostatic interactions and Van der Waals interactions). Hydrogels, also numerous functional hydrogels, are created, including biocompatible hydrogels, stimuli-responsive hydrogels, fluorescent nanocomposite hydrogels and hydrogels that promote self-healing. The crosslinked network enables the hydrogel to swell and absorb large amounts of water. The water absorption rate is closely related to the degree of crosslinking: the higher the degree of crosslinking, the lower the water absorption rate, and the hydrogels with good hydrophilic and flexible polymer networks can have excellent water absorption and retention capacity, which can expand rapidly in water, and in this swelling state can hold plenty of water without dissolving. The general state of a gel is neither a consummate solid nor a consummate liquid, whereas a solid has the property of being able to retain a certain shape and volume, and the behavior of a liquid is that it can diffuse or penetrate through the hydrogel.

This paper reviews the classification, principles and research progress of physical polymer gels based on hydrophobic bonding, hydrogen bonding, electrostatic and Van der Waals interactions, non-covalent bonding and polymer gels based on imine bonding, acylhydrazone bonding and dynamic chemical covalent bonding of a Diels–Alder reaction. The current situation and application prospects of polymer gel materials in oilfield drilling and production fields such as drilling fluids, fracturing fluids, enhanced oil recovery, etc. are summarized and sorted systematically to provide reference and inspiration for subsequent researchers.

## 2. Polymer Gel Material

A polymer gel is a three-dimensional mesh structure formed by the polymerization of molecular chains through crosslinking, or a mesh structure with a solvent (usually water), similar to biological tissue. The crosslinked structure makes it insoluble in water, and it maintains a certain form, such as a hydrogel. Due to the stimulation of different factors, such as type of polymer gel, salt concentration, pH, temperature, electrical stimulation and light irradiation [11,12,13,14,15], the volume of polymer gels undergoes certain changes, which are reversible and discontinuous. Several novel gels have been developed, such as temperature-sensitive gels, PH-sensitive gels, salt-sensitive gels, light-sensitive gels, shape memory gels and electric-field-corresponding gels [16].

### 2.1. Physically Crosslinking Polymer Gels

The physically crosslinking gel is a type of non-covalent bonding, which has the characteristics of reversibility and high performance [17]. Physically crosslinking gels are formed mainly by four non-covalent interactions: hydrophobic bonding interactions, hydrogen bonding interactions, electrostatic interactions and van der Waals interactions [18]. At normal physiological temperatures, interactions between non-covalent bonds are weak and transient, but multiple non-covalent bonds of different types act synergistically to form very stable and specific bonds, and physically crosslinked gels have the advantages of environmental friendliness, easy degradation, high shear resistance and dynamic reversibility compared to chemically crosslinking gels [19].

#### 2.1.1. Hydrophobic Association Interactions

**Figure 1:**
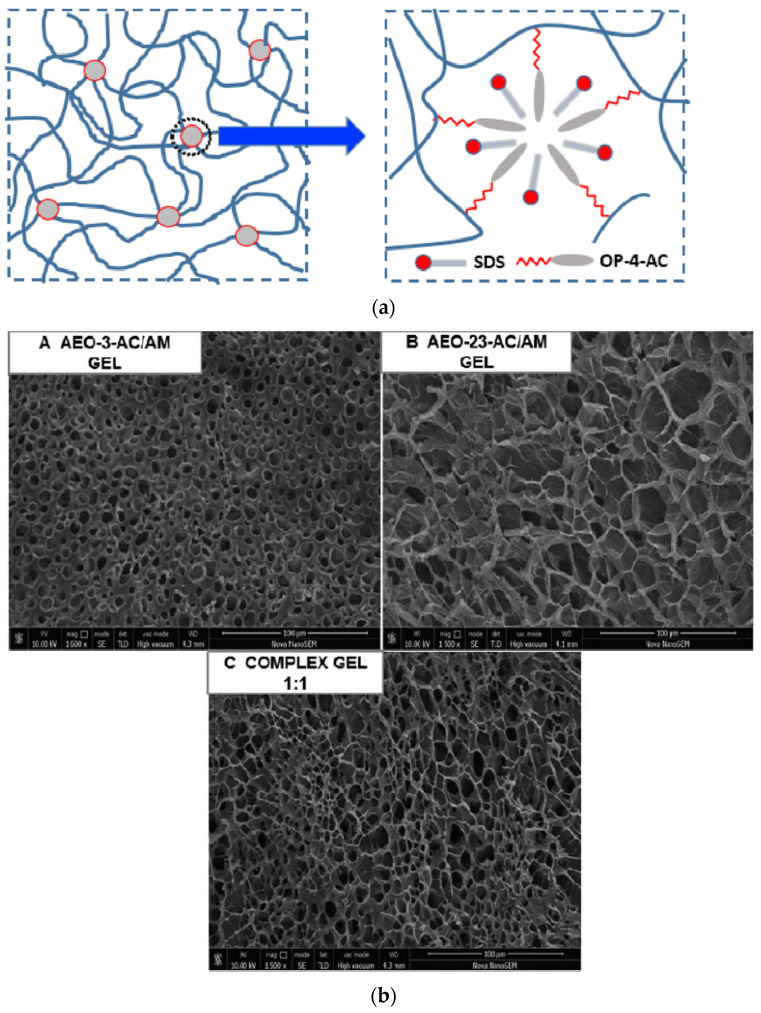
(**a**) Schematic diagram of the structural model of a hydrophobic-conjugated hydrogel with a conjugated network. (**b**) Scanning electron micrographs of hydrophobic-conjugated composite and non-composite gels [24].

Hydrophobic associative gels consist of crosslinking points of their three-dimensional networks by hydrophobic associative micro-regions, and this interaction between hydrophobic groups is dynamically reversible, so hydrophobic associative hydrogels have high mechanical strength, self-healing and secondary processing properties. The main methods for the synthesis of hydrophobic association gels are free radical copolymerization (micelle copolymerization, RAFT method) and parent chemical modification [20]. Generally, hydrophobic associative gels are obtained by micellar co-polymerization and comprise high amounts of hydrophilic monomers and low amounts of hydrophobic monomers depending on their hydrophobic association with hydrophilic units, hydrophobic units, surfactants and electrolytes. The hydrophobic monomers are bonded to form a micelle, which serves as a physically crosslinking point for hydrophobic association gels [21,22]. The hydrophobic association can be promoted by introducing short alkyl chains on the polyelectrolyte, and because the solubilization and bridging effect of the short alkyl chains, more hydrophobic areas of the association are formed, which leads to an increase in gel viscosity [23]. According to Liu et al. [24], the hydrophobic association gels were prepared by polymerizable amphiphilic monomers with a self-solubilizing compound micelle, and the SEM of the complex micelle was compared with the corresponding hydrogel SEM (Figure 1). It was found that the composition of the two types of amphiphilic monomers directly affects the micelle structure and micromorphology of the resulting gel. In addition, the mechanical properties of the composite hydrogel have been significantly improved compared to the corresponding non-composite hydrogel. Yang et al. [25] fabricated a hydrophobic and hydrogen-bonding-based supramolecular gel by micelle copolymerization, which can be used to seal cracks and pores in molds. The supramolecular gel is a crosslinking gel system with high-performance properties formed by non-covalent interaction during the self-assembly process. The rheological properties, mechanical properties, thermal stability and swelling ability of the supramolecular gels were investigated. The results show that the supramolecular gels have a dense three-dimensional network structure with open and interconnected pore structures and exhibit good rheological properties and high viscoelastic recovery. Taking environmental aspects into account, an environmentally friendly hydrophobic aggregation polymer was prepared by grafting long-chain alkyl groups onto xanthan gum via an esterification reaction, thus improving the oil production rate [26]. Hydrophobic association gel materials have the characteristics of high sensitivity, intelligent responsiveness and biocompatibility, but there is still much room for their development in raw material selection. The structural characteristics of hydrophobic association hydrogels determine their self-healing properties, but on a large scale, their self-healing process not only takes a long time, but also significantly decreases in strength after repair, and if electrostatic interaction, ligand interaction and weak bonding attached to the gel system, the repair time can be improved.

#### 2.1.2. Hydrogen Bonding Interactions

Hydrogen bonding is a common type of non-covalent bonding, usually weaker than covalent bonding; furthermore, it has the advantages of adjustability, addition and orientation, which can be enhanced by the synergistic effect of different hydrogen bonds [27]. Hydrogen bonding can confer many excellent properties to gels, such as self-healing, toughness and shape memory [28,29,30]. Hydrogen bonds typically form when a substance is in a liquid state, but in some crystalline and gaseous substances, hydrogen bonds can remain after they have formed [31].

**Figure 2:**
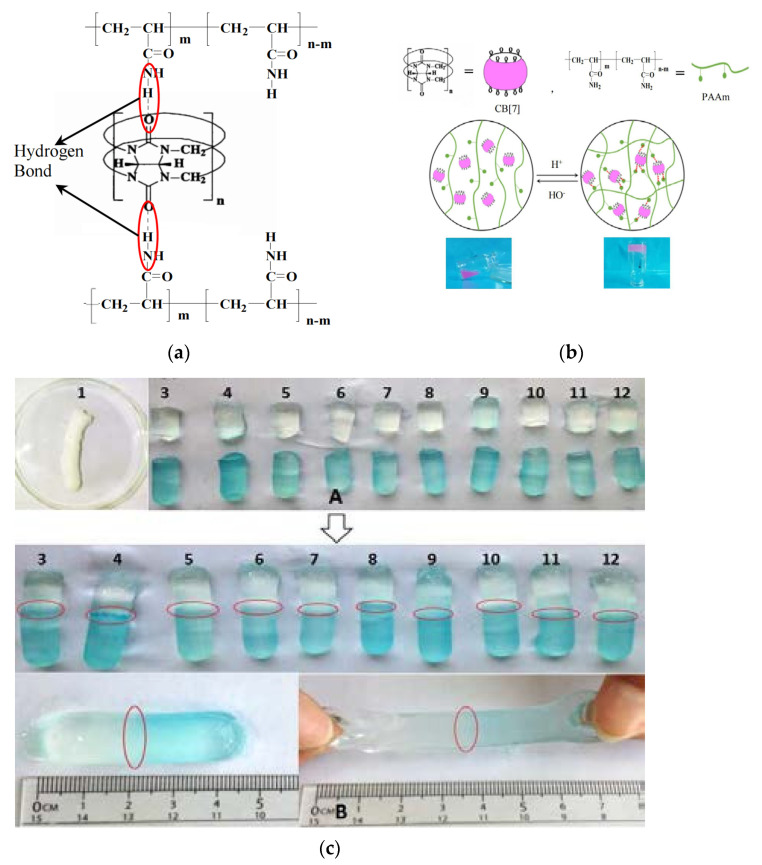
(**a**) Schematic molecular structure of CB[7]-PAAm gels. (**b**) Schematic diagram of the sol-gel phase transition of CB[7]-PAAm gels. (**c**) Effect of PH on the self-healing effect of CB[7]-PAAm gels(Red circles indicate gel self-healing joints) [33].

Curbituril (CB) is a macrocyclic compound similar to cyclodextrin (CD), and both have similar molecular properties [32]. The amino group of the polyacrylamide and the carbonyl group of the cucurbit are dissociated by hydrogen bonding (Figure 2), which is the main driving force for gel formation, and the solution–gel transition and self-assembly properties can be further controlled by the pH value [33].

**Figure 3:**
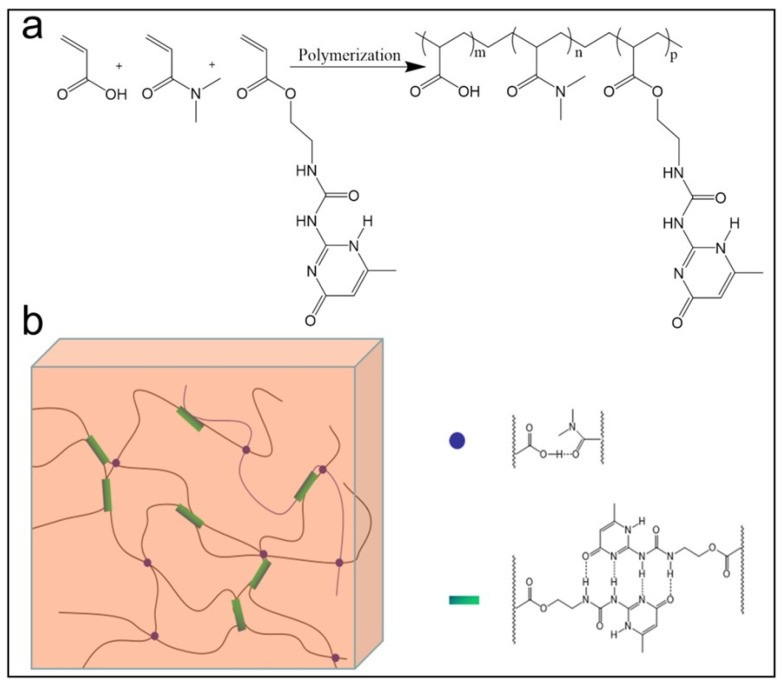
(**a**) Synthesis process and chemical structure of the hydrogel. (**b**) Demonstration of the presence of weak hydrogen bonds between acrylamide and acrylic acid and multiple strong hydrogen bonds between 2-ureido-4[1H]-pyrimidinone units in the hydrogel [35].

Hydrogels with only one hydrogen-bonding energy dissipation mechanism do not achieve satisfactory integrated mechanical properties, while multiple hydrogen bonds can achieve similar strength to covalent bonds or even stronger than covalent bonds. Li et al. [34] prepared a multiple hydrogen-bonded reinforced hydrogel with two types of hydrogen-bonded crosslinked regions: a hydrogen-bonded “soft” region and hydrogen-bonded “hard” region (Figure 3), this duo physically crosslinking a network of soft and hard synergistically (tensile strength of 2.34 MPa, elongation at break of 410%). This double, physically crosslinking network (tensile strength of 2.34 MPa and elongation at break of 410%) significantly enhances the mechanical properties of the gel, which also has good pH sensitivity because of multiple hydrogen bonding and can respond quickly to shape memory within a few minutes. A new type of hydrogel with multiple energy-dissipation properties has been developed using acrylamide, acrylic acid and a 2-ureido-4[1H]-pyrimidinone unit. Physical dissociation and reconnection of hydrogen bonds at different stress levels provide excellent mechanical properties for the hydrogel, and by optimizing the equilibrium between weak and strong hydrogen bonds, the transparent hydrogel has an adjustable Young’s modulus (70–1250 KPa) and a strong, stretchable height (up to 4340% strain) [35].

#### 2.1.3. Electrostatic Interactions

**Figure 4:**
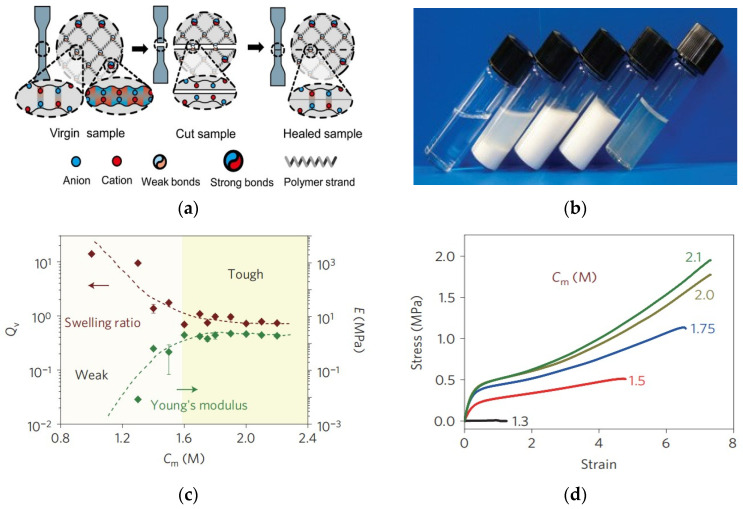
(**a**) Schematic diagram of the self-healing mechanism of polyampholytic electrolyte hydrogels. (**b**) Photographs of polyamphiphilic electrolytes polymerized with different total monomer concentrations. (**c**) Solvation volume ratio and Young’s modulus of amphiphilic electrolyte hydrogels with monomer concentration pair dependence. (**d**) Tensile behavior of polyamphiphilic electrolyte hydrogels with different monomer concentrations (The different color lines are the total molar concentrations of different ionic monomers) [37,40].

Electrostatically interacting gel is a type of gel that is formed by the copolymerizing of oppositely charged monomers in high concentration. The random charge distribution of polymorphic chains results in the formation of multiple ionic bonds within and between chains with fluctuating strength distributions [36]. The gels can be tuned by using different combinations of ions, thus changing a wide range of mechanical properties, and after dialysis of small counter ions, polyelectrolytes with opposite charges form polyionic complexes (PICs) with a wide range of power distribution, resulting in dynamic crosslinking over an extremely wide lifetime scale. The strength and weak ionic bonds enhance the material’s resistance to fracture and fatigue through the adhesive fracture, which enhances the material’s toughness. Weak links can also be used as reversible sacrificial links (Figure 4), providing high strength and self-healing for large deformations, forming ductile and viscoelastic gels with a variety of mechanical properties [37].

Electrostatically interacting gels are formed by reversible disruption and construction of strong and weak ionic bonds, and the prepared hydrogels also show responses to different stimuli (e.g., temperature, salinity and pH). In addition, since multiple shape memory effects can be achieved by relatively independent physical interactions in the gel, synthetic shape memory gels can be performed by programmable combinations [38]. Wang et al. [39] prepared hydrogel microspheres with porous and stable structures by hydrogen bonding and electrostatic interactions, and the porous structure of hydrogel microspheres was stacked by many planes, which made the pores more stable compared to chain networks. Hydrogel spheres have strong electronegativity because of the dehydrogenation of hydroxyl groups and, thus, have high affinity even at high pH values, making hydrogel microspheres stable and pleasant to use.

#### 2.1.4. Van der Waals Interactions

**Figure 5:**
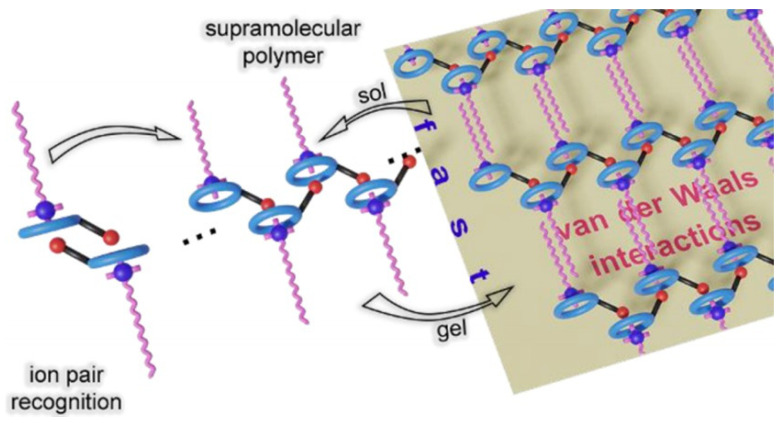
Sol–gel transition under van der Waals interactions [42].

Van der Waals interactions are an attractive force existing between molecules, which is much weaker than chemical bonding, but when a large number of van der Waals interactions are gathered, they also play a role in the self-assembly of gels as an auxiliary force. Van der Waals interactions are a kind of non-directional saturation that exists between molecules, and it has a decisive influence on the physicochemical properties of substances such as boiling point, melting point, the heat of vaporization, heat of melting, solubility, surface tension, viscosity, etc. Van der Waals interactions also play an important role in the formation of self-assembling structures [41]. Amharar et al. [42]. investigated whether the obtained supramolecular polymers showed the simultaneous occurrence of host–guest and cation-π interactions between carboxylic acids and cetyltrimethylammonium-functionalized cupro-lactones resulting in a rapid and reversible temperature-induced sol–gel transition because of van der Waals interactions of the cetyltrimethylammonium units (Figure 5).

Van der Waals interactions are among the intermolecular interaction forces, which are generated between nonpolar molecules or polar molecules through induced dipole interaction and can be divided into three specific forces: induced force, dispersion force and orientation force. Since it is difficult to form colloids by self-assembly through van der Waals interactions between molecules alone, several forces often interact together during gel formation. Many researchers usually combine hydrogen bonding, electrostatic interactions, and hydrophobic association with Van der Waals interactions to form higher-strength gels [43]. The thermal stability of the gel is improved by van der Waals interactions and the presence of intermolecular hydrogen bonds; moreover, the thermal stability of the gels in nonpolar and polar solvents is determined by the relative strength of the hydrogen bond between the molecules and the van der Waals interactions, which are weaker in polar solvents, and therefore, gelation occurs only when sufficient compensation is provided by integral van der Waals interactions [44].

### 2.2. Chemically CrossLinking Polymer Gels

Chemically crosslinking gels are a major class of polymer gels. It is formed by crosslinking in the form of covalent bonds between polymer chain segments and is non-spontaneous, where the polymer reacts with small molecule crosslinking agents (e.g., aldehydes, boronic acids) or the reaction is initiated by radiation (e.g., electron beam, γ-rays or UV light) [45]. Starting materials for chemically crosslinking gels can be monomers (water- or oil-soluble monomers), polymers or mixtures of monomer-polymers and have the advantages of better structural stability, relatively easy preparation and good modulation of physicochemical properties; however, crosslinking polymers break easily under the action of shear pumps and porous media, causing rapid and irreversible viscosity loss [46].

#### 2.2.1. Imine Bonding

Imine bonds are generated by the reversible condensation reaction of primary amines and reactive carbonyl groups (e.g., aldehyde and ketone groups), also known as the Schiff base, and the dynamic equilibrium of Schiff base bonds can be used to prepare self-healing hydrogels [47]. Dynamic imine bonds are PH-responsive, reversible and biologically active (Wolfel et al. [48]). Coexistence of 2-aminoethyl methacrylate hydrochloride (AEMA) with N, N′-diallylenediamide (DAT) in the same network can lead to self-healing hydrogels by introducing primary amino functional groups. Synthetic α-carbonyl aldehyde groups formed after the reaction of hydrogel and periodate form imine bridges near amine groups, and covalent imine bonds are formed spontaneously, resulting in the observed self-healing behavior of the gel.

**Figure 6:**
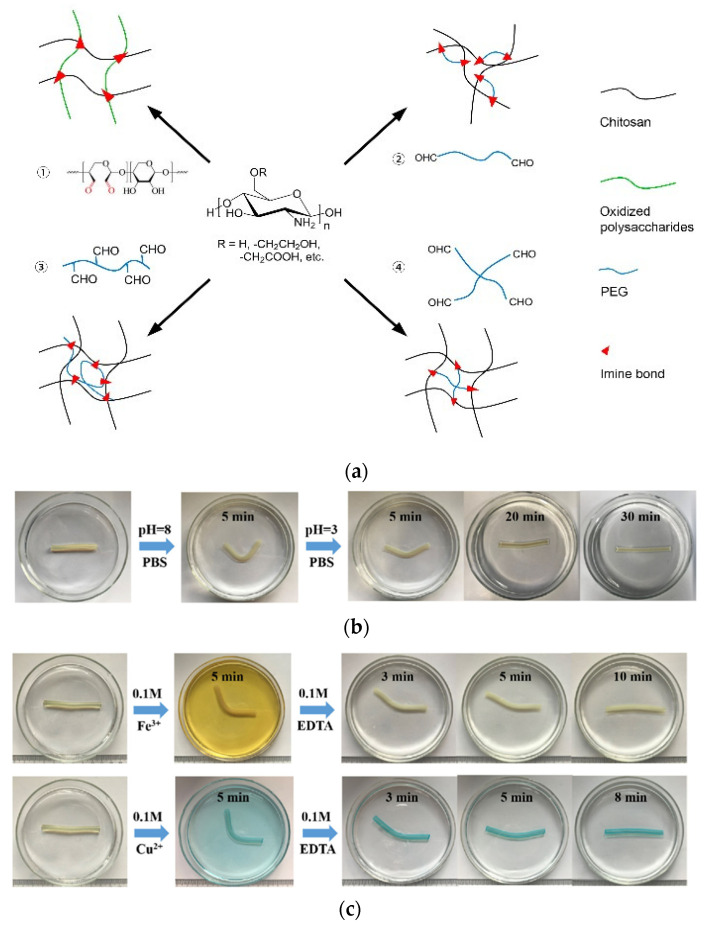
(**a**) Synthesis strategy of chitosan-based hydrogels. (**b**) Shape memory process of chitosan–polyacrylamide-based double network hydrogel in PBS solution (pH = 3). (**c**) Shape memory process of chitosan–polyacrylamide-based double network hydrogels in EDTA (0.1 M) aqueous solution [50,52].

Among these reversible chemical bonds, the imine bond has been widely applied, especially in chitosan-based self-healing hydrogels with numerous amino groups; in addition, a large amount of chitosan-based single-net hydrogel with a reversible dynamic imine bond has been developed. Chitosan, also known as diacetyl chitosan, is the only natural alkaline polysaccharide that can be used to produce self-healing gel materials [49]. Fu et al. [50] prepared hydrogels based on chitosan–polyacrylamide double-chain networks that were obtained by in situ radical polymerization (Figure 6). The hydrogel exhibited excellent mechanical properties: it was a self-healing and shape-sensitive material with a maximum tensile strength of 460 KPa and an elongation at a break of 4600%, respectively, while the elongation at break and strength at break of the hydrogel can be recovered under alkali stimulation at 35 °C because of the reversibility of imine bonding of 84.2% and 93.2%, respectively. Covalent bonding properties of free amino and carbonyl groups of chitosan and isopropyl benzaldehyde based on dynamic imine bonding formed gels with good mechanical properties, as demonstrated by rheological analysis, i.e., the energy storage modulus (G′) reached 107 Pa [51]. On the one hand, the self-healing ability of the gel is due to the dynamic equilibrium of the imine bonds, and on the other hand, the protonation of the chitosan amino group without crosslinking reaction, which confers the mobility of the chitosan molecular chain, is an important prerequisite for the self-healing properties while allowing the gel to exhibit PH-responsive properties.

#### 2.2.2. Acylhydrazone Bonding

The acylhydrazone bond is a Schiff base bond obtained by condensation of hydrazones with aldehydes (ketones) under weak acidic or weak basic conditions, which has better mechanical properties than the amine bond [53]. As a dynamic covalent bond, the acylhydrazone bond, although as stable as conventional covalent bonds in response to environmental conditions and as intelligent as supramolecular gels, presents a reversibility very different from other chemical covalent bonds; in addition, the crosslinking copolymers with dynamic acylhydrazone bond have a higher hydrodynamic radius and higher viscosity under high salinity conditions [54]. Qiao et al. [55] investigated whether alginate (ALG-ADH) modified with PEG (PEG-CHO) or hexanediol dihydrazide of aldehydes was used to form dynamic acylhydrazone bonds. Due to the dynamic acylhydrazone bond and multiple hydrogen bonds, the hydrogels exhibited excellent self-healing properties and the gels were able to retain 84.4% of their original elastic modulus. Chen et al. [56] used a dynamic double crosslinker in which acylhydrazone bonds (primary crosslinker) and borate ester bonds (secondary crosslinker) acted synergistically to form a multifunctional smart hydrogel with an excellent self-healing ability and high mechanical properties, with hydrogen bonds carrying the physical interaction.

**Figure 7:**
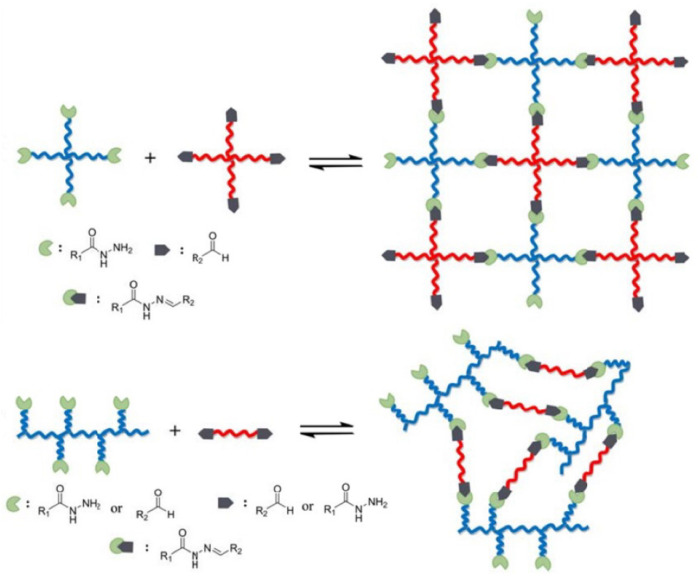
A model of dynamic covalent polymer networks crosslinking by acylhydrazone bonds (Different color lines indicate different polymer molecular chains) [57].

Dynamic covalent acylhydrazone bonds are very sensitive to environmental conditions, especially pH, and are unstable under acidic conditions; therefore, the rate of self-repair can also be regulated by pH (Figure 7). Under acidic conditions, the rate of hydrolysis and formation reactions results in the faster formation of network self-repair [57]. Therefore, Deng et al. [58] used a dynamic polymer hydrogel with atrazine and disulfide bonds as a single system characterized by environmentally adaptive self-reduction and dissolve-gel transition reactions, which self-heals under acidic (pH 3 and 6) and alkaline (pH 9) conditions by changing atrazine or disulfide bonds; however, the hydrogels could not self-heal at pH 7, but under the aniline catalytic effect, the self-healing ability is obtained by accelerating the exchange of acylhydrazone.

#### 2.2.3. Diels–Alder Reactions

**Figure 8:**
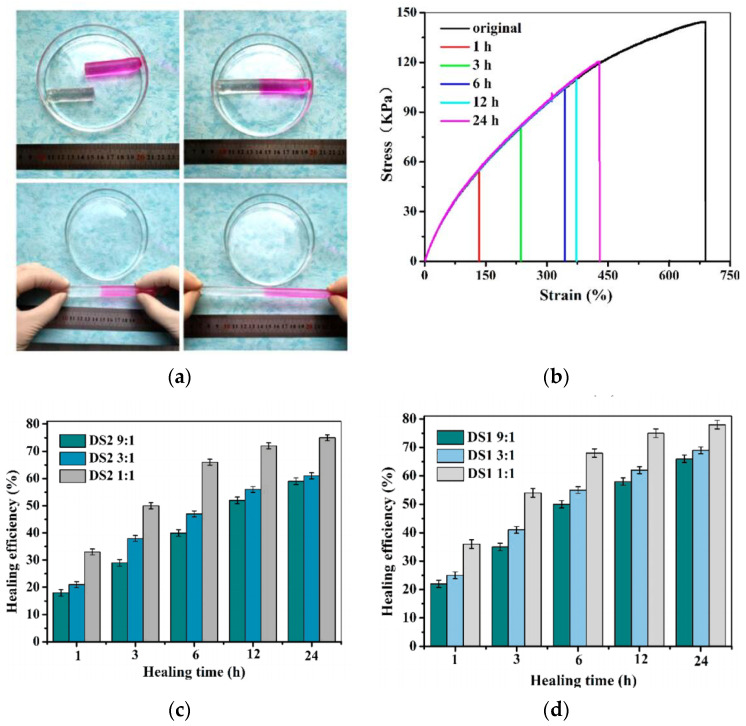
(**a**) Self-healing properties of hydrogels observed by direct visual observation. (**b**) Stress–strain curves of pristine and self-healing hydrogels at different healing times. (**c**,**d**) Self-repair efficiency of hydrogels tested by room temperature tensile [61].

The Diels–Alder (DA) reaction is a cycloaddition reaction involving conjugated dienes and parodies and is one of the most efficient synthetic methods for forming unsaturated extranuclear rings at low temperatures to create complex natural products, while the reverse reaction occurs at high temperatures [59]. Today, the most studied diene/dienophile system is furan/maleimide, which can be reversible in many cases above 100 °C via Diels–Alder cycloaddition reactions [60]. Shao et al. [61], by using Fourier transform infrared spectroscopy, confirmed the presence of a thermally reversible covalent click reaction between furan-modified CNC and maleimide-functionalized PEG and investigated a tough, high-modulus, fast self-healing nanocomposite hydrogel with excellent mechanical, self-healing and fatigue properties and evaluated its self-healing ability by tensile testing. The gel was found to have a self-healing capacity of up to 78% ( Figure 8). A series of self-healing pectin/chitosan hydrogels were prepared using the Diels–Alder reaction, which significantly increased the crosslinking density of the hydrogels and showed excellent self-healing properties in self-healing experiments and significant changes in swelling rates under different swelling environments, indicating good swelling properties, PH and temperature responsiveness [62].

**Figure 9:**
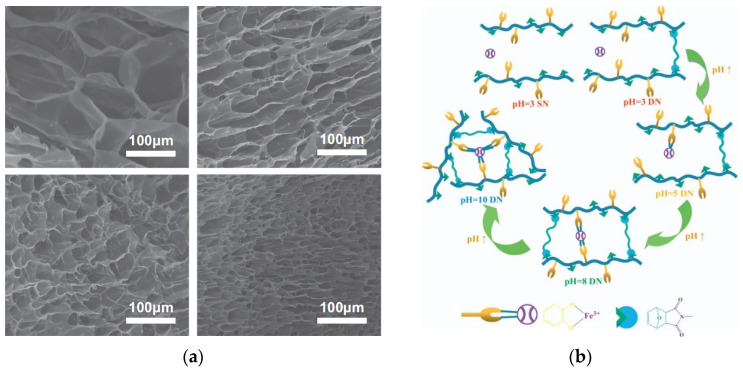
Crosslinking density and mechanical properties of DN hydrogels can be controlled by varying the ratio of Fe^3+^-catechol and pH value. (**a**) The pictures from left to right, top to bottom, are SEM of SN hydrogels; SEM of Fe^3+^-catechol 1:1DN hydrogels; SEM of Fe^3+^-catechol 1:2DN hydrogels; SEM of Fe^3+^-catechol 1:3 DN hydrogels. (**b**) Possible crosslinking mechanism of hydrogels at different pH conditions [63].

Dynamically chemically bonded self-healing gels established by the Diels–Alder reaction has mechanical properties superior to those of gels with dynamic imine and acylhydrazone bonds. To improve the mechanical properties of self-leveling gels, a series of dynamic chemical bonds or chemical–physical interactions must be developed [21]. Li et al. [63] used a DN hydrogel with excellent thermomechanical and self-repairing properties that was designed and manufactured by Diels–Alder reaction and coordination effect. In addition, by adjusting the proportion of Fe^3+^-catechol, the mechanical, swelling and surface morphology of DN hydrogel can be controlled. The Diels–Alder click and coordination effect of smart hydrogen generated by Diels–Alder click and coordination guides the design of novel hydrogels with superior mechanical properties, self-healing properties and controllable crosslinking density (Figure 9).

## 3. Application of Functional Polymer Gels

### 3.1. In Drilling Fluids

Drilling fluid is a general term for circulating fluids that perform a variety of functions required by the drilling process. From conventional to unconventional, deepwater to ultra-deepwater, as oil fields are explored, the technological challenge of drilling fluid is becoming more and more serious [64]. The commonly used polymer gels can be divided into subsurface crosslinking gels and surface pre-crosslinking gels. Because of the forming conditions, the synthesis principle and the preparation method of the two gels are different, and the differences in temperature, salt and chemical resistance are also different.

For fractured strata, conventional sealing materials have insufficient dimensions and low strength, leading to first-time seal failures and increased costs [65]. The major advantage of subsurface crosslinking polymer gel is that it can produce high-strength block gel in the pores and cracks of the formation to realize a high-strength sealing zone. To deal with severe leakage or great leakage, and to meet the need for plugging and pumping at different depths of the well, it is necessary to stop drilling and plugging using subsurface crosslinked gels. Weak gels are three-dimensional crosslinked systems consisting primarily of inter- and intramolecular crosslinkers, complemented by low concentrations of polymers and weak crosslinkers. As the concentration of crosslinkers increases, the network structure of the weak gel becomes more uniform and stronger (Huang et al. [66]). The formation of weak gels with a spatial network structure by hydrogen bonding of polar amide groups in nonpolar solvents can make the emulsions exhibit excellent shear thinning and thixotropy, which substantially improves the gel strength and solves the problems of low yield point, rock carrying and poor borehole cleaning ability. To overcome the lack of salt resistance of water-based drilling fluids, Li et al. [67] used free-radical polymerization to synthesize weak gels, which exhibited special rheologic properties and enhanced salt resistance. The weak gels have a 3D mesh structure, and with the increase in the concentration of the weak gels, the hydrophobicity between the molecules is increased, and the network structure is more compact, which can suppress the dispersion and swelling of the clay.

**Figure 10:**
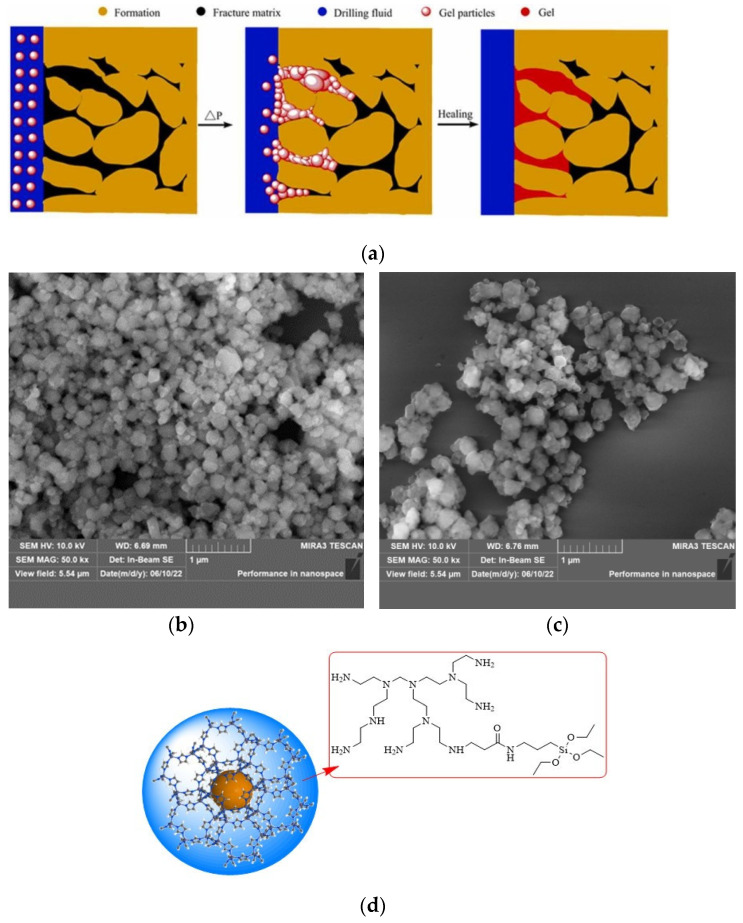
(**a**) Plugging process of polymer self-healing gel. (**b**) SEM of modified ZIF particles. (**c**) SEM of modified ZIF particles after ageing. (**d**) Schematic diagram of the structure of modified ZIF [69,70].

Polymer gel plugging agents are usually polyacrylamide-based gel systems formed by polymeric crosslinking metals or organic crosslinkers, such as pre-crosslinking gel particles (PPG), polymeric microspheres, dispersion particle gels (DPG) and other ground pre-crosslinking gels. The main advantages of prefabricated crosslinked gels are that they can accommodate pore and crack sizes and are not affected by solidification temperatures, salinity, and shear. Fan et al. [68] developed a fractured-formation polymer plugging gel particle; the covalent bonding of the reactive carboxylate and hydroxylate monomers used as raw materials improves the physical properties of the gel. The gel also has good deformation capacity; with a pressure-bearing capacity of up to 21 MPa and counterpressure capacity of up to 20 MPa, it is more than twice as strong as conventional polymer buffer gels and can significantly improve formation pressure capacity and well control (team of academic Sun Jinsheng [69]). A new type of core-shell gel particle (modified ZIF) was prepared (Figure 10) that possessed both rigid and flexible sealing properties and crosslinks with sulfonated phenolic resin (SMP-3) and sulfonated lignite resin (SPNH) in drilling fluids to increase the viscosity of the fluid as it passes through the gaps in the barrier layer formed by the modified ZIF particles, preparing insoluble gels and gel complexes of modified ZIF particles to increase the long-term thickening properties of the drilling fluid, successfully adjusting the rheological properties of the drilling fluid and optimizing the efficiency of reducing seepage losses.

### 3.2. In fracturing Fluids

Hydraulic fracturing in oil wells is a very important technological tool for reservoir modification and is the first method used to increase production from oil and gas wells. The main objective of this method is to inject fluids containing all types of proppants into the wellbore at high flow rates and pressures. Fracturing fluids are an important component of fracturing and need to be refined and improved to meet the requirements of complex fracturing reservoirs [71].

**Figure 11:**
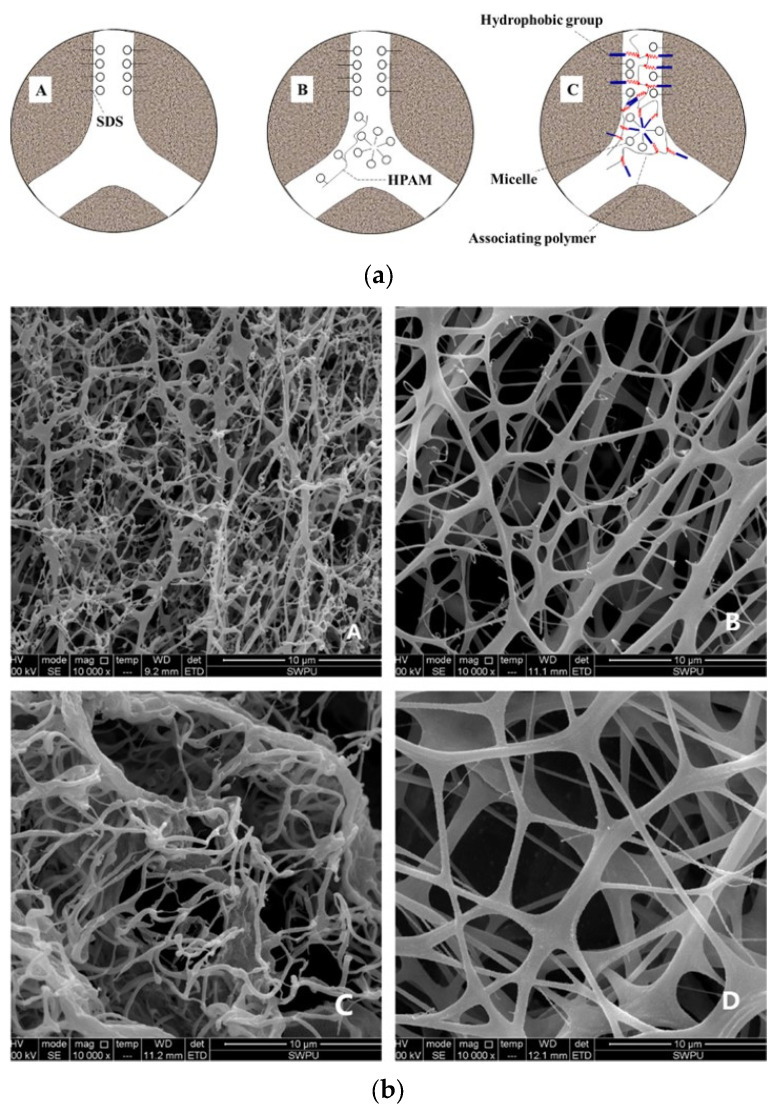
(**a**) Schematic diagram of the effect of surfactant on the stability of A surfactant with polyacrylamide, B surfactant with the associative polymer and C foam. (**b**) A and B are ESEM images before and after the addition of SDS to the solution of the associative polymer; C and D are ESEM images before and after the addition of crosslinking agent to the solution of guar gum [73].

Depending on the kind of polymer used, polymer-based fracturing fluids are divided into biopolymer, synthetic polymer and composite systems. Guar gum chains enable biopolymers to resist high-temperature environments through hydrogen bonding high molecular weight, extensive intermolecular linkage in aqueous solutions and the use of high-temperature resistant additives (organometallic crosslinkers and gel stabilizers). Boric acid crosslinkers can be synthesized by optimizing the properties of boric acid and crosslinkers by adding ester bonds to the molecular structure and increasing the amount of boric acid to improve the stiffness and flexibility of the coordination bonds, resulting in a very-low-concentration rubber-shredding fluid that can withstand temperatures up to 175 °C, which can be used to solve the problems of conventional aggravated fracturing fluids such as large amounts of gum breakers and difficulties in rejection [72]. Xiong et al. [73] developed a new type of foam fracturing fluid; Figure 11b shows the microstructural changes in the solution before and after crosslinking with the guar melt. Before crosslinking, the molecular chains were layered, and there was no three-dimensional network structure, but after adding boron, a dense network was formed between the cis-hydroxyl groups of boron and guar gum, which improved the stability and viscoelasticity of the foamed washing solution.

In oil extraction, fracturing is a very important technology for low-permeability reservoirs. Because of the complexity of rock geology, the conventional acid fracturing fluid cannot satisfy the technical requirements for the exploitation of carbonate resources. A new type of acid gel fracturing fluid can be prepared under non-covalent interaction, which has ultra-high viscosity, excellent sand-carrying performance and good salt resistance and can maintain high viscosity and good rheology under high mineralization conditions and exhibit good high-temperature stability, which makes up for the shortage of conventional fracturing fluid performance at the present stage and can meet the needs of increasing production in unconventional low-permeability shale reservoirs [74]. To improve the performance of fracturing fluids in highly mineralized, high-temperature reservoirs, Fan et al. [75]. developed gel systems with rigid hydrophobic-conjugated spatial network structures and linear entanglement to improve the degree of thickening, solubility, temperature adaptability and sensitivity to the chemical environmental resistance of the polymer. The copolymer fluids were found to exhibit high creep-recovery performance, elastic response, 100% viscosity recovery, temperature stability and salt resistance through shear sensitivity tests (Zhao et al. [76]). Through the process of self-assembly of hydrophobic compounds to form network structures, the new fractionated solution exhibits better propane and butane suspension properties than crosslinking hydroxypropyl gum under static conditions. The anionic surfactant also improves the physically crosslinking network by forming mixed aggregates of hydrophobically bound micelles, solving the salt resistance problem of conventional polymer fractionated solutions and demonstrating low structural friction and high sand-transport capacity in field tests.

### 3.3. In Enhanced Oil Recovery

With most mature oil fields entering the development stage of high water content and low oil production, injecting water-plugging agents into deep formations to expand the wave volume and chemically drive oil to improve recovery is the most common technical solution [77]. Ordinary gels are prone to mechanical degradation in the formation; their viscosity decreases significantly, and they have poor sealing ability for high permeability channels. The properties of weak gels are between those of dispersion gels and native gels, which have a moderating effect in deep reservoirs and further in enhanced oil recovery [78]. The presence of Cr^3+^ induces crosslinking reactions between different chains of the same molecule in weak gels, resulting in crosslinking polymer systems with localized mesh structures, which have a high resistance to flow but can still flow, thus allowing deep conditioning [79]. In the development and application of water diversion technology in low permeability reservoirs, Cui et al. [80] studied the crosslinking kinetics of low water-soluble HPAM/phenolic gel systems with optimized viscosity, HPAM and binder that can achieve high thermal stability and shear strength and, based on nuclear explosion experiments, demonstrated residual oil in the displacement process It was demonstrated that residual oil in the small pores forms large oil droplets that migrate into the oil stream during the displacement process, demonstrating that the weak gel improves oil recovery in low-permeability oil layers.

**Figure 12:**
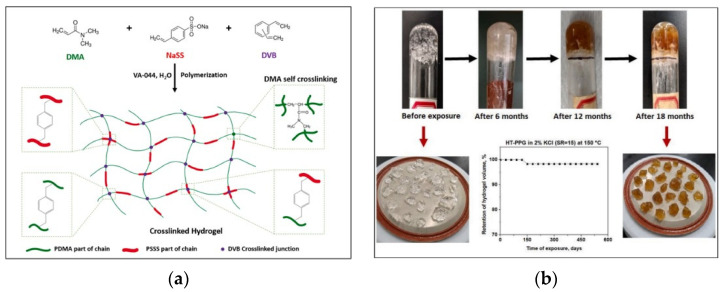
Ultra-high-temperature-tolerant hydrogels (HT-PPGs). (**a**) General synthetic scheme for HT-PPG hydrogel synthesis. (**b**) Thermal stability evaluation of HT-PPG in 2% KCl at SR = 15 and 150 °C. HT-PPG recovered after 18 months of ageing and showed retention of structural integrity and gel particle morphology [84].

The deeper the oil and gas reservoir is buried, the higher the reservoir temperature, and the adaptive microgel (SMG) as a deep-seated dissection agent is limited by the complex pore structure and the strict matching of particle size. The poor mechanical properties and the non-degradable characteristics seriously limit its application. In response to some problems of current gel materials as chemical drives applied in oil fields, the main deficiencies are a too-fast water absorption rate, temperature resistance and poor long-term stability under high mineralization. Polymer nanoparticles are very flexible in controlling the blocking effect, and after high-temperature dissolution, the high-permeability layer was selectively blocked, and the low-permeability layer was hardly blocked, so polymer nanoparticles can be better applied to deep transfer drive technology [81]. Liang et al. [82] synthesized high-viscosity nano gel particles by a radical reaction combining the action of gel and nanoparticles and used a dispersion of high-viscosity nano gel particles at 250 mPas at 90 °C to close the channel and remove the remaining oil droplets, achieving about 30% recovery of light and dark oil in a core propulsion experiment. The commonly used dissection agent preformed granular gels (PPGs), PPGs show good temperature and salt resistance and the expansion rate of PPGs is kept above 10 at a 70 °C or 130 °C (10,000 mg/L NaCl solution) temperature; however, PPG with larger particle size may have a greater blocking effect. It is difficult to achieve deep dissection, and the validity period during dissection is short [83]. For improvement of polymer mechanical performance based on temperature and salt resistance, Salunkhe et al. [84] prepared a hydrogel (HT-PPGs) with ultra-high temperature tolerance. HT-PPGs can dissolve more than 30 times their initial volume in brines with different ionic strengths and have good mechanical properties, with storage modulus (G′) exceeding 3000 Pa at 90% water content. in addition, HT-PPGs exhibit excellent hydrolysis at 150 °C in 2% KCl brine (when the water content is greater than 93%). C showed excellent hydrolytic thermal stability for more than 18 months (Figure 12). It shows good sealing efficiency in core replacement tests and helps to reduce the permeability of fractures, which is ideal for use in reservoir transfer drives to enhance recovery under harsh temperature and salinity conditions.

## 4. Research Prospect of Functional Polymer Gels

### 4.1. In Drilling Fluids

As oilfield exploration and development continue, construction will be more difficult, especially for deep wells, complex wells and special process wells and for the development of special reservoirs, and with the importance of environmental protection, the requirements for drilling fluids are becoming higher and higher, and resistance to high-temperature, high-pressure, complex formations in deep wells, and oil and gas formation protection are still important directions for the development of drilling fluid technology. Conventional drilling fluids can adsorb to the clay surface through electrostatic attraction or hydrogen bonding to prevent clay swelling, but cationic polymers are incompatible with drilling fluids and are easily lost to adsorption to the clay particle surface, while ionic polymers are less resistant to high mineralization rates and have lower surface adsorption strength and are believed to provide superior pore consolidation in water-sensitive formations. Therefore, PH-responsive drilling fluids and high-temperature, low-temperature, high-pressure and environment-friendly drilling fluids need to be vigorously researched.

With the development of oil and gas drilling to unconventional, ultra-deep and ultra-high temperature reservoirs, well leakage management will also become more and more difficult. The poor compatibility of common plugging materials with the formation and the weak mechanical properties of plugging gel cause a poor one-time plugging effect and easy-to-occur repeated leakage. Although the surface pre-crosslinking gel can be plugged with drilling, its low pressure-bearing capacity is not suitable for larger fracture strata, while the underground crosslinking gel can seal larger fractures, but it needs to stop drilling to seal, and the gel particles take longer time to become gel and delay the drilling period [21]. Therefore, it is urgent to develop new high-strength polymer gels for good leak plugging by optimizing the crosslinking density to improve the stability of the gels.

**Figure 13:**
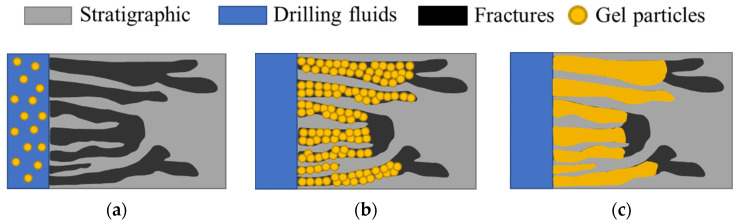
Mechanism of polymer self-healing gel plugging. (**a**) Gel particles circulate in the wellbore with the drilling fluid. (**b**) The gel particles are adsorbed and accumulated in the formation of fractures. (**c**) The gel particles are extruded and deformed to form a block gel to seal the fracture.

Since the mechanical properties of the gel made by multiple hydrogen bonding crosslinking are strong, and there is reversible destruction and construction between strong and weak ionic bonds in electrostatic action; the multiple hydrogen bonding action and electrostatic action are introduced into the gel material, and the polymer chains are connected by physical entanglement, thus forming a three-dimensional structure with enhanced strength (i.e., viscosity and elasticity) between the dense structure of the crosslinking local area. After the gel particles enter the formation with drilling, the particles will be adsorbed in the formation fractures under the action of hydrogen bonding and electrostatic force, and with the continuous accumulation of gel particles, under the action of formation pressure, the gel particles will produce self-healing phenomenon after water absorption and expansion, so that the particles will condense into a block with high mechanical properties (Figure 13), which improves the pressure-bearing capacity of the formation, thus realizing long-term effective sealing of the fractures.

### 4.2. In Fracturing Fluid

Oil and gas output may be increased by using a procedure called hydraulic fracturing. This process’s main objective is injecting the propellant slurry into the wellbore at high flow rates and pressures. This process can increase the permeability of the reservoir by fracturing the rock to form permanent fractures in the well [85]. Deep unconventional reserves are now being explored and developed for oil and gas. Higher demands are placed on the performance of fracturing fluids. Guar gum fracturing fluid enables polymers to resist high-temperature environments below 150 °C through hydrogen bonding high molecular weight, the utilization of high-temperature-resistant chemicals and substantial intermolecular bonding (organometallic crosslinkers and gel stabilizers). However, conventional guar gum fracturing fluids suffer from large amounts of insoluble residue and incomplete gel breaking, reducing the permeability of the proppant required for oil and gas flow.

**Figure 14:**
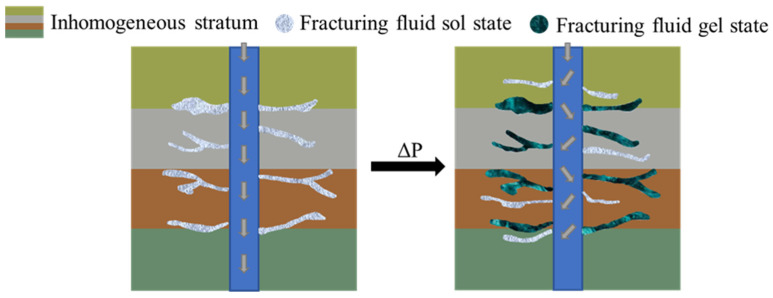
Schematic diagram of the sol–gel conversion mechanism of polymeric fracturing fluid.

The non-covalent interaction between hydrophobic association, hydrogen bonding and electrostatics provides the synergistic mixed-phase system with high-temperature resistance. The use of silica nanoparticles as the base point increases the physically crosslinking density by the crosslinking polymerization method, which leads to the rapid growth of the strength of the hydrogel and makes it a better and more stable three-dimensional structure. When the polymer gel enters the formation fracture after extrusion, the gel’s mechanical properties are improved and a dynamic reversible sol–gel phase transformation is produced, which can be used as a fracturing fluid material (Figure 14). Therefore, from the viewpoint of the structural design of polymer material, it is necessary to vigorously develop high-temperature-resistant crosslinking agents with higher crosslinking efficiency, as well as high-temperature stability and lower-cost polymer gel fracturing fluids to meet the trend of oil and gas development in the future.

### 4.3. In Enhanced Oil Recovery

With the rapid development of the petroleum industry, oil and natural gas exploration has expanded and many fields have entered a medium to high development phase. Facing the serious problem of large channel development, when the water content of producing wells exceeds a certain amount, it has a great impact on the production of oil wells. Many large-scale dominant channels are formed in the deep part of the reservoir, resulting in serious inefficient and ineffective circulation, decreasing oil production and rising water content too fast in oil wells. Depth dissection technology is one of the most effective ways to improve the wave and volume of injected fluid and control inefficient and ineffective circulation [86,87]. The deep dissection will encounter a vicious environment of high temperature and high mineralization, and to solve the above problems, it is necessary to study the temperature and salt-resistant dissection and water plugging agent for low permeability and high-water-bearing wells to improve the recovery rate.

**Figure 15:**
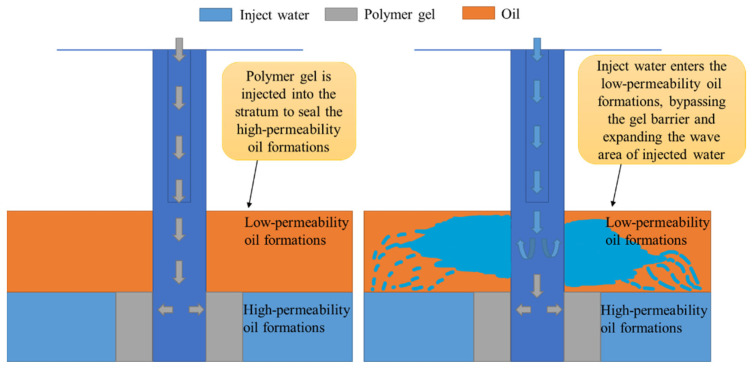
Polymer gel for water conditioning and blocking mechanism.

The polymer gel prepared by polyacrylamide and organic chromium, Cr^3+^, first hydrates to form a six-ligand hydration ion, then forms a double bond bridge ligand through hydrolysis and hydroxyl bridge, further polymerizes to form a multinuclear hydroxyl bridge complex ion, and then crosslinks with -COO- in the polymer polyacrylamide chain link to form a temperature- and salt-resistant gel structure, and silica nanoparticles are used to fill gaps in the 3D gel networks to keep them stable in high-salinity reservoirs, thereby maintaining their stability in high-salinity reservoirs, which has good temperature and salt resistance because of its special molecular structure and makes the crosslinking polymer system reversible and the viscosity recoverable by introducing acylhydrazone bonds. The use of polymer gel for water drive after conditioning makes the injection water flow into the low permeability reservoir preferentially, bypassing the gel barrier and increasing the injection wave area to achieve stable injection control, which effectively improves the injection development effect and increases the enhanced oil recovery (Figure 15).

Polymer gel has good environmental responsiveness and can meet the requirements of sand carrying, support and filtration loss reduction of a high-performance fracturing fluid, but it is less applicable to the formation of malignant environments because of its application in the field of oilfield development, such as a weak gel chemical drive to improve enhanced oil recovery, etc. Therefore, more attention needs to be paid to the research and development of ultra-high-temperature, ultra-high salt and PH-responsive gel.

## 5. Conclusions

(1) Polymer gel material is a three-dimensional mesh structure formed by the polymerization of molecular chains through crosslinking. The classification of the crosslinking mode is divided into physically crosslinking gels with hydrophobic bonding interaction, hydrogen bonding interaction, electrostatic interaction and van der Waals interaction and chemically crosslinking gels with imine bonding interaction, acylhydrazone bonding interaction and Diels–Alder reaction. The reversible changes in the molecular structure of the gels through covalent or non-covalent bond interactions have strong mechanical properties. Therefore, environmentally responsive gels and self-healing gels can be developed for bioengineering, oil field development, artificial intelligence, etc.
(2) Polymer gel materials are suitable for drilling fluids, fracturing fluids and improving the enhanced oil recovery. As oil fields are increasingly exploited at depth, more attention is paid to the development of high-temperature and salt-resistant polymer gels. Weak gels, plugging gels and drilling fluid materials such as well-wall reinforcing agents enter the formation with drilling and adsorb in the fractures and seal and strengthen the good wall after water absorption and expansion, extrusion and deformation and other external forces. Polymer gel is used as a fracturing fluid in the formation to create a seal by sol–gel conversion and increase the permeability of the formation. In the field of chemical oil drive, polymer gel increases the enhanced oil recovery by temporarily sealing the high permeability layer so that the injected water flows into the low permeability reservoir preferentially and increases the wave area of the injected water.
(3) As oilfield extraction is increasingly moving to high-temperature and ultra-deep wells, the exploration environment is becoming more and more hostile, so the polymer gel materials needed also need to be temperature resistant, salt resistant and PH responsive and to possess other properties. We promote the development of an intelligent polymer gel and a higher-performance self-healing gel for malignant leak plugging, well wall stabilization and recovery enhancement work. In addition, we expand the application fields of polymer gel materials, develop scientific and intelligent working methods and promote the development of polymer gel materials in the direction of more intelligence.

## Data Availability

Data sharing is not applicable to this article.
